# Accuracy of facial skeletal surfaces segmented from CT and CBCT radiographs

**DOI:** 10.1038/s41598-023-48320-0

**Published:** 2023-11-28

**Authors:** Mohammed Ghamri, Konstantinos Dritsas, Jannis Probst, Maurus Jäggi, Symeon Psomiadis, Ralf Schulze, Carlalberta Verna, Christos Katsaros, Demetrios Halazonetis, Nikolaos Gkantidis

**Affiliations:** 1 Department of Orthodontics and Dentofacial Orthopedics, School of Dental Medicine, University of Bern, 3010 Bern, Switzerland; 2 Jeddah Second Health Cluster, Ministry of Health, Riyadh, Saudi Arabia; 3 Department of Oral and Maxillofacial Surgery, School of Dentistry, National and Kapodistrian University of Athens, 11527 Athens, Greece; 4 Division of Oral Diagnostic Sciences, Department of Oral Surgery and Stomatology, School of Dental Medicine, University of Bern, 3010 Bern, Switzerland; 5 Department of Pediatric Oral Health and Orthodontics, UZB-University Center for Dental Medicine, University of Basel, 4058 Basel, Switzerland; 6 Department of Orthodontics, School of Dentistry, National and Kapodistrian University of Athens, 11527 Athens, Greece

**Keywords:** Anatomy, Translational research

## Abstract

The accuracy of three-dimensional (3D) facial skeletal surface models derived from radiographic volumes has not been extensively investigated yet. For this, ten human dry skulls were scanned with two Cone Beam Computed Tomography (CBCT) units, a CT unit, and a highly accurate optical surface scanner that provided the true reference models. Water-filled head shells were used for soft tissue simulation during radiographic imaging. The 3D surface models that were repeatedly segmented from the radiographic volumes through a single-threshold approach were used for reproducibility testing. Additionally, they were compared to the true reference model for trueness measurement. Comparisons were performed through 3D surface approximation techniques, using an iterative closest point algorithm. Differences between surface models were assessed through the calculation of mean absolute distances (MAD) between corresponding surfaces and through visual inspection of facial surface colour-coded distance maps. There was very high reproducibility (approximately 0.07 mm) and trueness (0.12 mm on average, with deviations extending locally to 0.5 mm), and no difference between radiographic scanners or settings. The present findings establish the validity of lower radiation CBCT imaging protocols at a similar level to the conventional CT images, when 3D surface models are required for the assessment of facial morphology.

## Introduction

One of the primary goals of craniofacial radiology research is to develop accurate and efficient imaging techniques for the skeletal facial structures. For example, researchers may investigate the use of advanced imaging modalities, such as computed tomography (CT) or cone beam computed tomography (CBCT), along with superimposition techniques, to create a realistic virtual patient model^1^ or to better visualize and quantify changes in complex craniofacial structures over time^2,3^. These 3D imaging techniques may be preferrable to conventional 2D techniques (e.g., lateral cephalograms), as they provide a more complete understanding of the spatial relationships between different structures and are not affected by limitations inherent to 2D imaging, such as magnification, distortion, and overlap of neighbouring structures^4^.

The image quality, and thus, the diagnostic value of a 3D radiograph can greatly vary depending on the radiographic machine, the image acquisition parameters (voxel size, kV, mA), the field of view (FOV) and the complexity or consistency of the scanned object, especially in CBCT images^2,5,6^. CBCT is the most common 3D radiographic examination in dentistry and offers generally reduced radiation exposure and faster acquisition time than a conventional CT scan, at the expense of image quality^7^. Another shortcoming is the usage of grayscale values, instead of the standardized grey values (Hounsfield units) that a CT offers. In addition, CBCTs present higher noise and scatter level, as well as geometric errors, as opposed to the CT images^8^. This information is crucial for proper diagnosis, treatment planning, and outcome assessment, especially in cases where the skeletal anatomical form is of high importance.

Normally, to use the entire 3D anatomical form of a structure, skeletal surface models need to be segmented from the original radiographic volumes. This can serve several purposes, such as the evaluation of morphological changes occurring as a result of pathology, growth, or treatment^9,10^ or the construction and 3D printing of individualised appliances^11^. However, apart from the acquisition parameters, previous studies show that bone segmentation, especially from CBCT images, can also affect the derived surface models^2,10^, jeopardizing potentially valuable 3D information.

The accuracy of the radiographically derived skeletal surface models is fundamental for several disciplines^5,10,12^. Accuracy is the combination of trueness and precision. Trueness refers to the closeness of an extracted model to the true skeletal facial surface and precision refers to the degree of consistency and reproducibility of repeatedly extracted surface models under the same conditions (ISO 5725-1). Living human studies are not suitable for this purpose, due to ethical considerations related to patient exposure to radiation^13^. Repeated exposures, as well as the accurate depiction of the actual anatomy, is not possible in vivo, since this would require unjustifiable radiation doses. In vitro or ex vivo study designs are the only feasible methodological approaches aiming to adequately represent actual clinical conditions. Ex vivo experimentation on dry cadaveric specimens is often used to validate X-ray imaging techniques^14,15^. However, the absence of soft tissues affects the resulting image compromising the applicability of outcomes in actual patients^16^. This is usually addressed by embedding the dry skeletal specimens in soft-tissue simulants during radiographic imaging, such as water, wax sheets, or gel-like materials^17,18^. Previous studies on the accuracy of radiographically derived skeletal surface models from CT or CBCT images either lack soft-tissue simulation^16,19^ or compare surface models hydrated by soft-tissue simulants to directly scanned dry specimens through high-accuracy optical surface scanners^14,15^. The latter approach is also problematic since it does not consider the effect of hydration on the anatomical form of the skeletal specimens^20,21^.

The accuracy of CBCT imaging in depicting the 3D facial skeletal surface has not been adequately tested so far. Thus, the primary objective of this study was to identify the effect of different machines, radiation dose settings, and segmentation thresholding on the validity of the segmented skeletal surface models of the face from CT and CBCT radiographs. For this purpose, CT and CBCT skull data originating from different acquisition configurations were investigated, with direct surface scans of the specimens through an optical scanner serving as the gold standard.

## Materials and methods

### Material

The material consisted of ten human dry skulls collected from the Municipal cemetery of Serres, Greece, following the required approval from the local authorities (Municipality of Serres, Greece, Protocol Number: 4044/12.07.2018) (Supplementary Fig. 1). This was performed in the context of a large project investigating 3-dimensional superimposition techniques on skeletal structures of the head^20,22^. As reported previously^20^, all handling of human tissues was compliant with the relevant local legislation. The specimens belonged to humans that were deceased between 8 and 12 years prior to the study implementation. At the time of acquisition, there were no claims from any relatives and the identity of the specimens was not known. Hence, informed consent was not pursued, as the ethics committee granted a waiver for it. Our goal was to choose intact skulls of adult size, free from any indications of significant aging or pathology. The sample size was arbitrarily defined based on data and resource availability and the authors’ research experience. According to our previous study, a minimum sample of eight specimens was considered adequate^23^, but we decided to increase the sample size to ten to facilitate the statistical analysis and obtain more robust findings^22^.

### Image acquisition

All skulls were directly scanned in hydrated conditions^20^ with a structured-light, 3D surface optical scanner and were also subjected to CT and CBCT imaging in similar conditions. The radiographic images were obtained at hydrated conditions for soft-tissue simulation. The same conditions were created for the direct surface scans to account for the effects of hydration on dimensional integrity^20^ and ensure comparability.

The dry skulls were embedded in tap water for approximately 15 min. The water was in room temperature (22–25 °C) and had a pH of approximately 7.5. Afterwards, they were removed from water, they were gently patted with tissue paper, and were immediately scanned using a high-accuracy, optical 3D surface scanner (Artec Space Spider, Artec3D, Luxembourg; Software: Artec Studio 12, Version 12.1.6.16). Prior to scanning, the scanner was calibrated according to the manufacturer’s instructions. The complete outer facial surface was the primary target, although the complete image of the skull was acquired. The entire sample preparation and scanning process has been published previously and showed high precision at about 40 μm^20,22^. The subsequent 3D surface models were used as gold standard models for the study.

**Figure 1:**
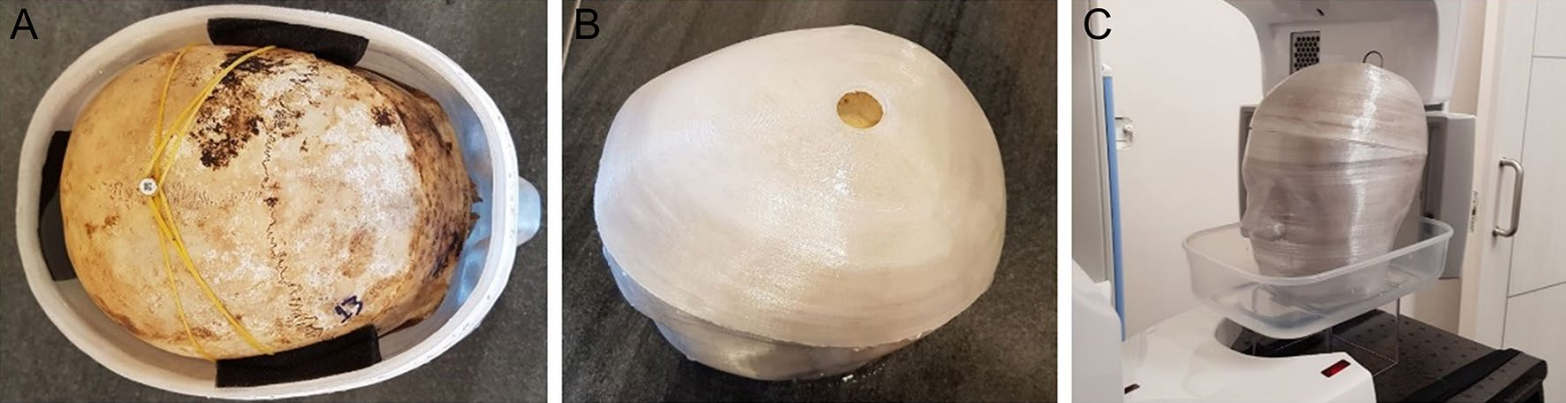
(**A**). Final configuration of an entire skull subjected to radiographic imaging. (**B**) The 3D printed soft-tissue head shell was filled with water, through the hole present at its upper part. (**C**). Radiographic image acquisition with soft-tissue simulation.

Within a few days, CT and CBCT images were obtained from the same specimens, with soft tissue simulation achieved by enclosing each specimen in a 3D printed head shell (PETG, MasterFill Premium PETG Pro, 3DHUB, Greece), filled with water^17,18,24^. The entire head bony specimen was centered in the head shell, using radiolucent water resorbing sponges (Fig. 1). Three head shells of different size were designed in a way to accommodate every specimen, while allowing for realistic soft-tissue thickness between the shells’ inner wall and the outer surface of the specimens.

Each specimen underwent four full head radiographic scans using the following acquisition settings:

1. CT machine (Revolution CT 256, GE Healthcare; 251 Hellenic Airforce Hospital, Athens, Greece). kV: 120, mA: 490 in the area of interest (automatically configured based on tissue mass and density), exposure time: 1 s, slice thickness: 0.625 mm, voxel size: 0.49 to 0.62 × 0.49 to 0.62 × 0.31 (interslice) mm, FOV: full head (displayed FOV: 25 cm).
2. CBCT machine I (Newtom VGiMK4, Verona, Italy; Dental School, National and Kapodistrian University of Athens Greece). kV: 110, mA: 4–5 (automatically configured based on tissue mass and density), exposure time: 4 s, voxel size: 0.3 × 0.3 × 0.3 mm, FOV: 15 × 15 × 15 cm.
3. CBCT machine II—regular dose settings (Planmeca F, Planmeca Promax 3D Mid 2018; Digital Iatriki Apeikonisi, Athens, Greece). kV: 100, mA: 8, exposure time: 12 s, voxel size: 0.2 × 0.2 × 0.2 mm, FOV: 20 × 20 × 20 cm.
4. CBCT machine II—ultra-low dose settings (Planmeca U). kV: 100 kV, mA: 8, exposure time: 6 s, voxel size: 0.2 × 0.2 × 0.2 mm, FOV: 20 × 20 × 20 cm.

All radiographic scans were performed by professional radiologists under standardized conditions regarding the water embedding process and the anatomical form stability of the specimens. Corresponding slices from radiographic volumes of one skull, through each acquisition setting, are provided in Supplementary Fig. 2.

### Surface model generation

#### Optical surface scanner data

The raw data of the directly scanned skulls through the surface scanner were semi-automatically post-processed in the Artec Studio 16 software (Version 16.0.5.114, Luxembourg, Luxembourg) to create a complete full head surface model. The surface models were saved as STL and OBJ file formats. The detailed 3D model generation process has been published previously and showed very high precision ranging from 5 to 10 μm^22^.

All 10 surface models that were acquired through surface scanning were considered as the gold standard reference. These were imported in Viewbox 4 software (dHAL Software, Kifisia, Greece) for comparison with the corresponding radiographically acquired surface models, to test the trueness of the latter.

#### Radiographic data

The CT and CBCT radiographic images were exported in DICOM format and imported in Viewbox 4 to extract the facial surface models through a visually defined single threshold segmentation process. One experienced operator (MG) assessed all models and selected the optimal threshold through gradual adjustment. To facilitate this process, image contrast was enhanced by removing the irrelevant extreme greyscale values from visualization. Afterwards the threshold isoline was adjusted manually to best conform on the outer skeletal surface edge on several 2D radiographic slices. The final threshold was the one that, after potential adaptation, provided the best segmentation of the entire facial model, according to the visual assessment of the operator. Each selected threshold was recorded in a Microsoft Excel sheet (Microsoft Corporation, Redmond WA, USA). The subsequent dense triangular mesh models, which were generated in Viewbox 4 software using a variant of the marching cubes algorithm^25^, were exported and saved as STL files. They consisted of approximately 900,000, 1,600,000 and 4,000,000 vertices, for CT, Newtom, and Planmeca unit-generated volumes, respectively.

### Measured outcomes and surface model superimposition

#### Intra and inter-operator reproducibility of the visually defined segmentation threshold

The visual segmentation process of all radiographs was repeated by the same operator (M.G.) at least 2 weeks following the first extraction to test intra-operator reproducibility. A second operator (M.J.) with experience in single threshold identification repeated the entire process for 16 randomly selected radiographic volumes (four from each setting), following a calibration session with the first operator. The selected threshold values were recorded in a Microsoft Excel sheet and compared.

#### Intra-operator reproducibility of the visually segmented surface models

**Figure 2:**
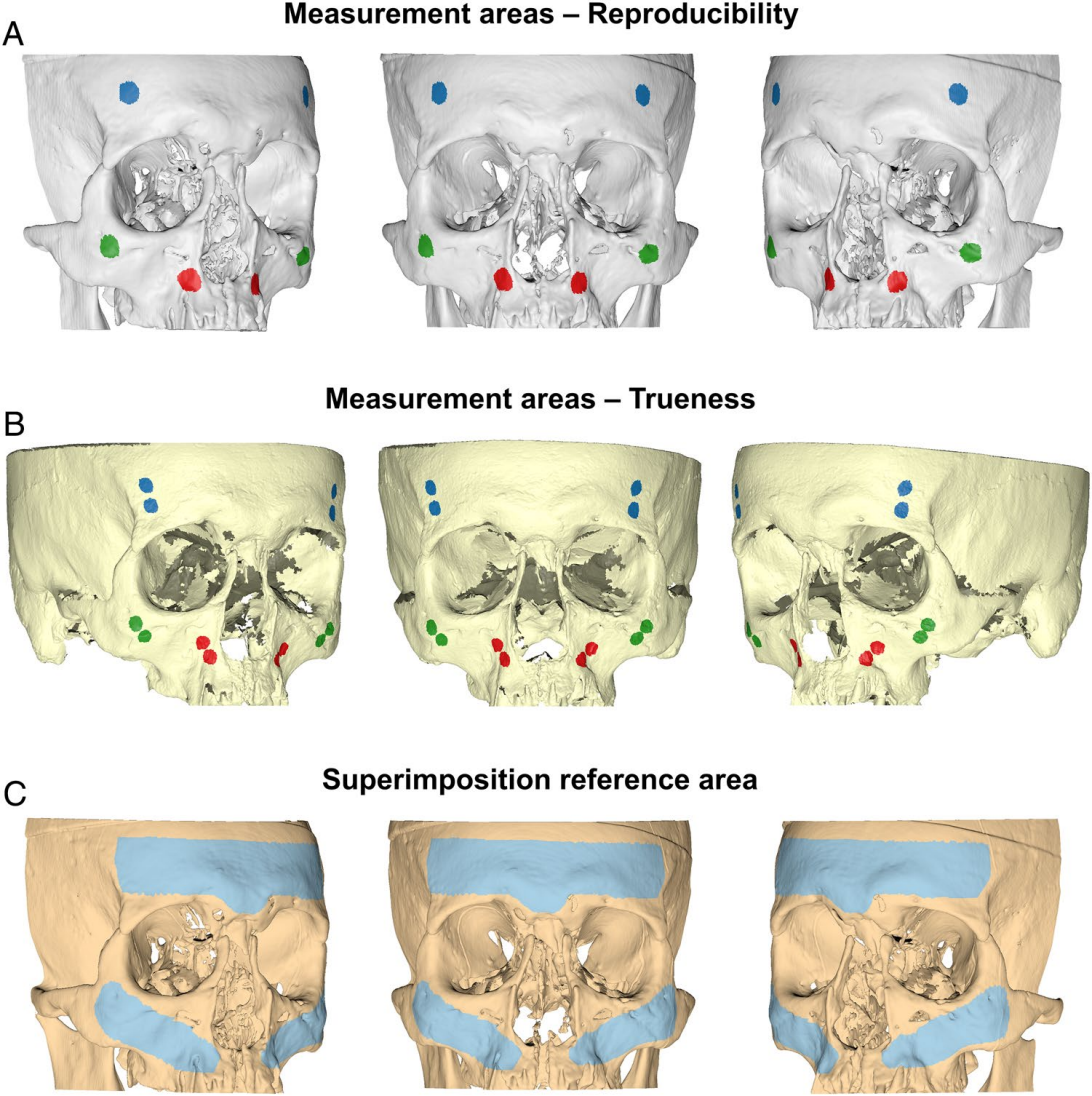
(**A**, **B**). Three measurement areas defined in each skull through bilaterally selected mesh surfaces at the forehead (blue), the zygomatic process (green), and the maxillary process (red) for reproducibility and trueness assessment, respectively. Each circular area shown in the images consists of 1000 triangles. (**C**). Reference area (light blue) used for all surface-based superimpositions performed in the study.

Intra-operator reproducibility of the visually segmented surface models was tested at three levels, similarly to a previously applied method^2^. First, the mean absolute distance (MAD), as well as the standard deviation of the absolute distances (SDAD), of the repeatedly extracted models was calculated, considering the distance of each vertex point of one mesh model to the closest point on the second model, at three predefined measurement areas consisting of 2000 triangles each. For this assessment, the segmented surface models retained their spatial relations in the source radiographic volume. The measurement areas were located on the forehead, the zygomatic process, and the maxillary complex, bilaterally. The bilaterally selected triangles on each anatomical structure were considered as one measurement area (Fig. 2A). Colour coded distance maps between repeatedly segmented entire models were generated to represent the cases with minimum, average and maximum difference on all measurement areas. Afterwards, each pair of repeated models was superimposed through a variant of the iterative closest point (ICP) algorithm^26^, under the following software settings: 100% estimated overlap of meshes, matching point to plane, exact nearest neighbour search, 100% point sampling, 50 iterations. The used superimposition reference area is shown in Fig. 2C. The rotational and translational movements required for the best fit approximation of each pair of models were recorded to describe differences between their original position and the position after superimposition. Finally, the MAD (SDAD) between the superimposed models at the pre-defined measurement areas was calculated to test their morphological differences, independent of their position in space. Zero MAD prior to superimposition and zero movements were considered as perfect reproducibility.

#### Trueness of radiographically derived surface models

The trueness of the radiographically derived skeletal surface models was tested through comparison with directly obtained models using a high-accuracy optical surface scanner^20,22^. For this purpose, each radiographically derived surface model was best-fit approximated to its corresponding optical scanner derived model, using the same settings and reference areas described above, regarding the intra-operator reproducibility of the visually segmented surface models. The congruence of the superimposed models on three pre-defined measurement areas, consisting of 4,000 triangles each, was calculated as MAD (SDAD) and this was the metric to assess trueness. The measurement areas were placed bilaterally on the forehead, the zygomatic process, and the maxillary complex. The bilaterally selected triangles on each anatomical structure were considered as one measurement area (Fig. 2B). These measurement areas were selected only once for each skull, on the optical scanner derived surface, and were used for all comparisons to eliminate confounding due to measurement area selection. Colour coded distance maps between superimposed entire models were generated to represent the cases with minimum, average and maximum difference on all measurement areas. Zero difference between the superimposed models indicates perfect trueness.

### Statistical analysis

The IBM SPSS statistics for Windows (Version 28.0. Armonk, NY: IBM Corp) was used to produce relevant box plots and perform descriptive and comparative statistical analyses.

Data were tested for normality using the Shapiro–Wilk test and though the visualization of normality plots and significant deviations were detected in certain variables. Thus, non-parametric statistics were applied.

Differences between repeated identifications of the visually defined segmentation threshold were tested through Wilcoxon signed rank test and shown in box plots. Differences between the four acquisition settings were tested using Kruskal–Wallis test. The overall difference in the amount of error was tested through Mann–Whitney U test.

Differences in intra-operator reproducibility of the visually segmented surface models between the four acquisition settings were tested using Kruskal–Wallis test, followed by Mann–Whitney U test for pairwise comparisons (significance values adjusted by the Bonferroni correction), if significant differences were detected by the first. These tests were performed once considering all measurement areas as one variable and once considering each measurement area as a single variable.

Differences in the trueness of radiographically derived surface models by the four acquisition settings were tested in a similar manner.

### Ethical approval and informed consent

Ethical approval for the project was obtained from the research ethics committee of the Dental School of the National and Kapodistrian University of Athens (Protocol number: 335, Date of approval: 02/05/2017, Renewed on 16.11.2021). All methods were performed in accordance with the relevant guidelines and regulations.

## Results

### Intra and inter-operator reproducibility of the visually defined segmentation threshold

**Figure 3:**
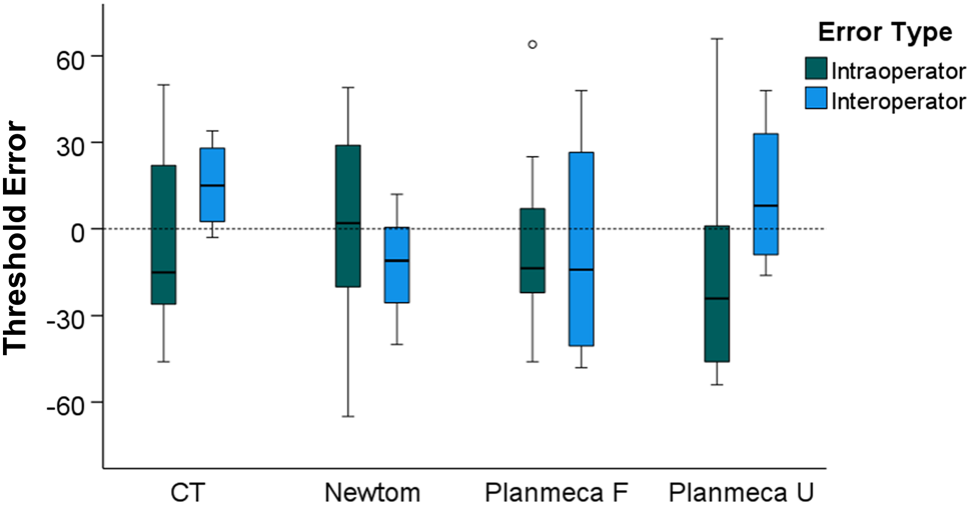
Box plots showing the intra- and inter-operator differences in visually defined facial surface segmentation threshold values for radiographic volumes. Outliers are shown as black circles (further from the median more than 1.5 times the IQR). A difference of 10 in threshold values corresponds to 0.25% of the full range of voxel values of the CT images, 0.22% of the Newtom images, and 0.29% of the Planmeca images.

The median difference between repeated, visually defined segmentation threshold values by the same operator was small (median: − 9.0, IQR: 47.5; Wilcoxon signed rank test: *p* > 0.05) and did not differ between machines (Kruskal–Wallis test: *p* = 0.622). Comparable outcomes were evident for inter-operator differences (median: 1.5, IQR: 35.7; Wilcoxon signed rank test: *p* > 0.05; Kruskal–Wallis test: *p* = 0.410). Overall, when all differences between repeatedly defined thresholds were considered as one variable, similar amounts of error were evident within or between operators (Mann–Whitney U test: *p* = 0.364; Fig. 3).

### Intra-operator reproducibility of the segmented surface models

Intra-operator reproducibility of the visually segmented surface models was high, with the maximum MAD being 0.067 mm (SDAD: 0.026 mm) and 0.027 mm (SDAD: 0.023 mm), before and after superimposition of the repeatedly segmented models, respectively. Both were detected in the maxillary process depicted in a CT scan for MADs and in a Newtom scan for SDADs.

**Figure 4:**
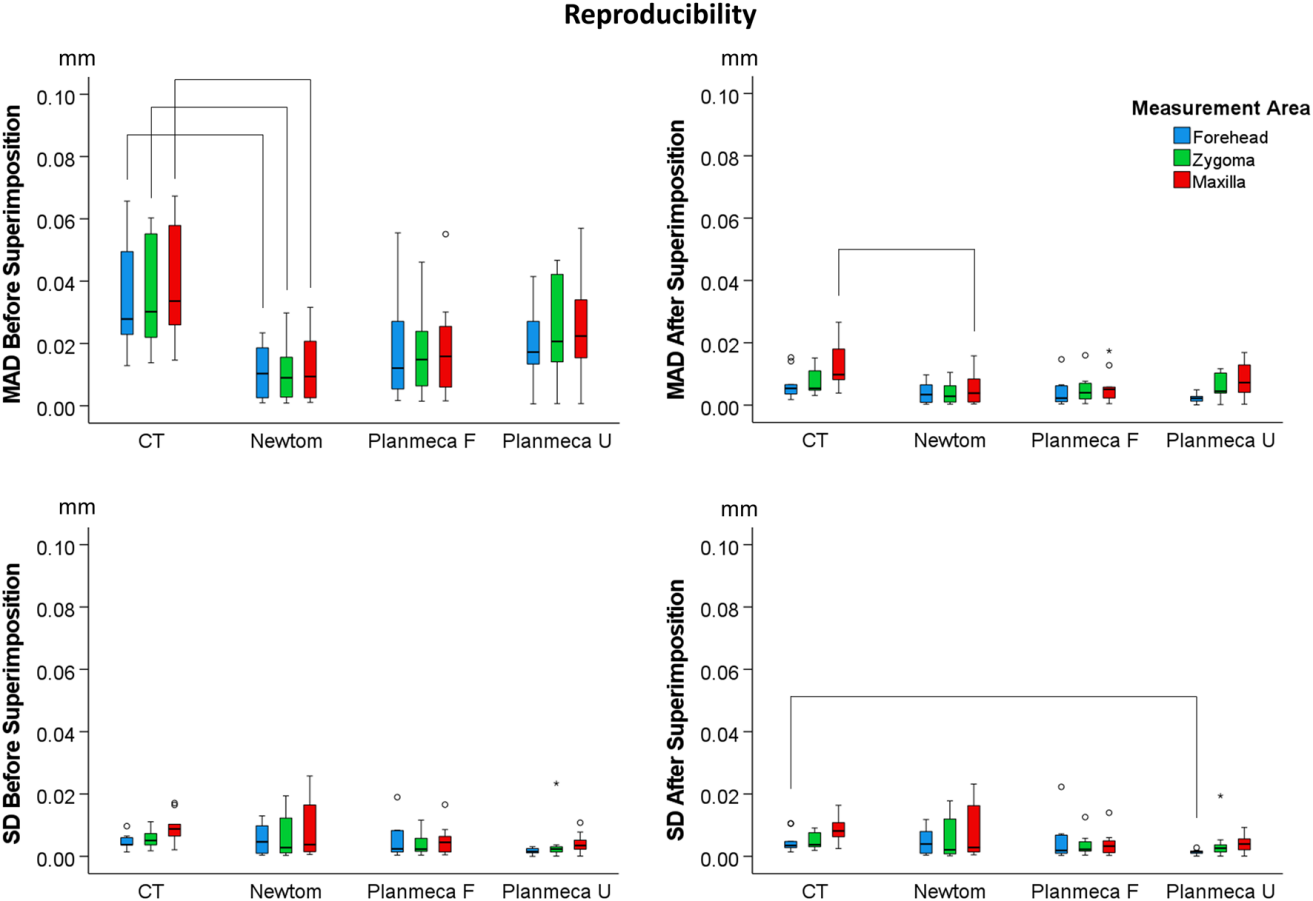
Box plots showing the intra-operator differences between repeatedly segmented facial surface models from radiographic volumes. The upper graphs show Mean Absolute Distances (MAD) between the corresponding surface models and the lower graphs the Standard Deviations of the absolute distances (SD). The lines connect variables that show significant differences (*p* < 0.05) detected through Kruskal–Wallis, followed by Mann–Whitney U test (Bonferroni adjusted). Outliers are shown as black circles (further from the median more than 1.5 times the IQR) or asterisks in more extreme cases (further from the median more than 3 times the IQR).

There was a significant difference between acquisition settings in reproducibility (MAD), considering all measurement areas as one variable (Before superimposition: *p* < 0.001, pairwise comparisons Newtom vs. Planmeca U: *p* = 0.023, Newtom vs. CT: *p* < 0.001, CT vs. Planmeca F: *p* < 0.001; After superimposition: *p* = 0.002, pairwise comparisons Newtom vs. CT: *p* = 0.006, CT vs. Planmeca F: *p* = 0.024). Considering the position of the extracted models in space (MAD before superimposition), the CT derived models showed the highest differences between repeated segmentations (median: 0.030, IQR: 0.032 mm), followed by Planmeca U (median: 0.020, IQR: 0.020 mm), Planmeca F (median: 0.014, IQR: 0.020 mm) and Newtom (median: 0.009, IQR: 0.017 mm). Significant differences were also evident for the SDADs between the repeatedly extracted models (Before superimposition: *p* = 0.001, CT vs. Planmeca U: *p* = 0.001; After superimposition: *p* = 0.003, CT vs. Planmeca F: *p* = 0.045, CT vs. Planmeca U: *p* = 0.005). Before superimposition, the CT derived models showed the highest SDADs between repeated segmentations (median: 0.006, IQR: 0.005 mm), followed by Newtom (median: 0.004, IQR: 0.009 mm), Planmeca F (median: 0.003, IQR: 0.005 mm), and Planmeca U (median: 0.002, IQR: 0.002 mm) (Fig. 4).

When each area was analysed as a single variable, Newtom showed consistently better reproducibility (MAD) than CT in all areas prior to best-fit approximation of the models (Forehead: *p* = 0.008, Zygoma: *p* = 0.008, Maxilla: *p* = 0.005). After approximation of the models, this outcome remained significant only for the maxilla (*p* = 0.038). Regarding the SDADs of the repeated models, before and after superimposition, all comparisons showed no differences, except from one (CT vs. Planmeca U: *p* = 0.038) (Fig. 4).

**Figure 5:**
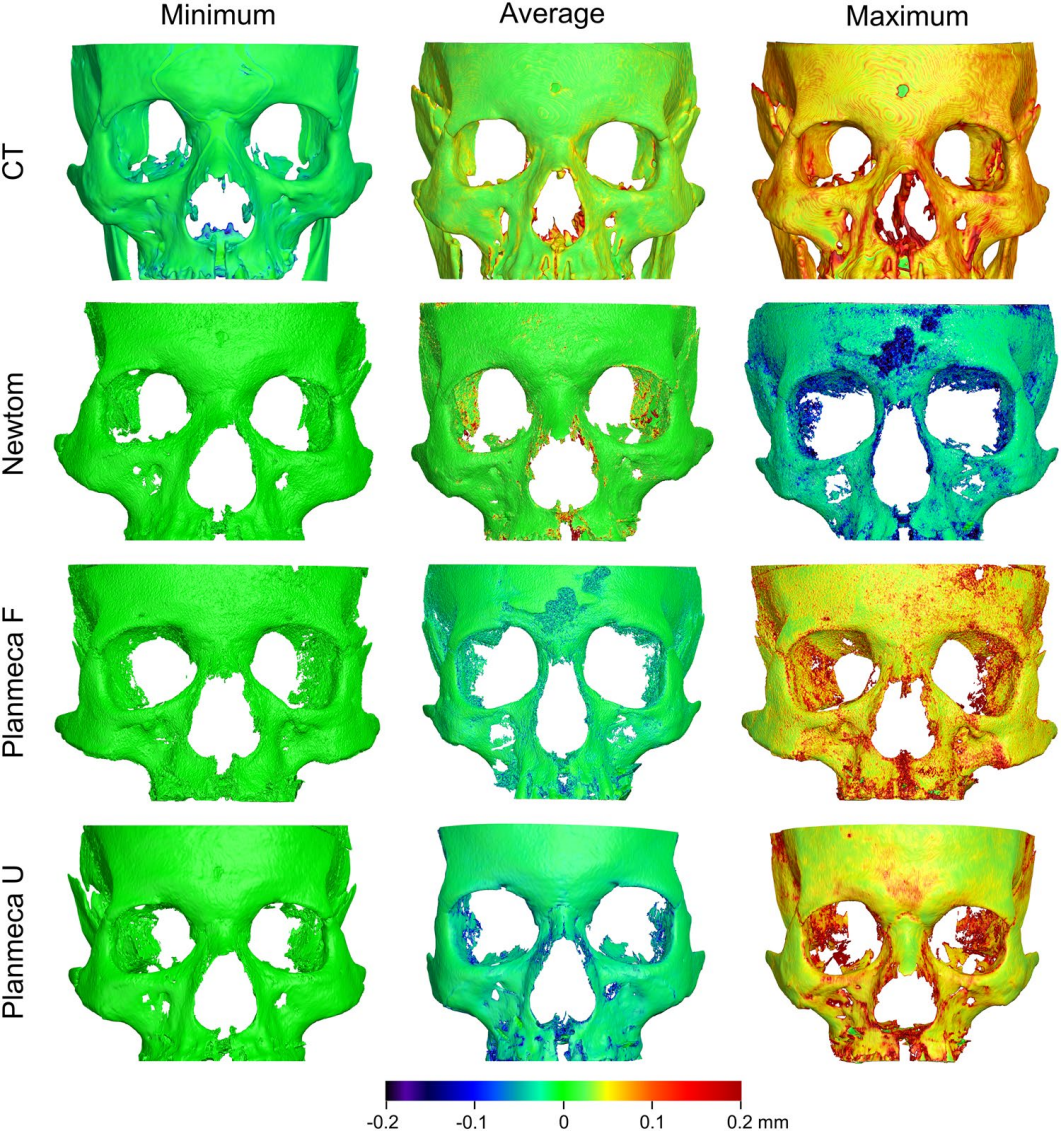
Colour coded distance maps between repeatedly segmented surface models by the same operator, representative of the minimum, average, and maximum differences detected in the sample for each acquisition setting. The compared models retained their original spatial relation within the source radiographic volume (status before superimposition).

For all acquisition settings, colour coded distance maps between repeatedly segmented surface models, at their original position, revealed uniformly distributed differences, limited within a range of 0.1 mm (Fig. 5).

**Figure 6:**
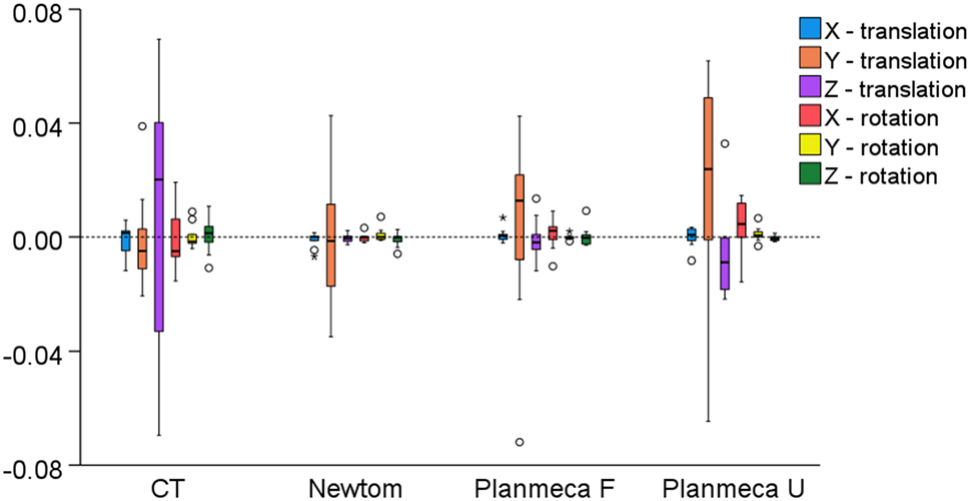
Box plots showing the rotational (°) or translational (mm) movements required to best-fit approximate the repeatedly segmented surface models by the same operator, for each acquisition setting. Outliers are shown as black circles (further from the median more than 1.5 times the IQR). X-translation: lateral movement, Y-translation: vertical movement, Z-translation: anteroposterior movement, X-rotation: around the lateral axis, Y- rotation: around the vertical axis, Z- rotation: around the anteroposterior axis.

There was no difference between acquisition settings in any rotational or translational movement required to best-fit approximate repeatedly segmented surface models by the same operator, (X-translation: *p* = 0.608, Y-translation: *p* = 0.263, Z-translation: *p* = 0.431, X-rotation: *p* = 0.354, Y-rotation: *p* = 0.145, Z-rotation: p = 0.501). The magnitude of translation or rotation was very limited, consistently less than 0.08 mm or degrees, respectively (Fig. 6).

### Trueness of radiographically derived surface models

**Figure 7:**
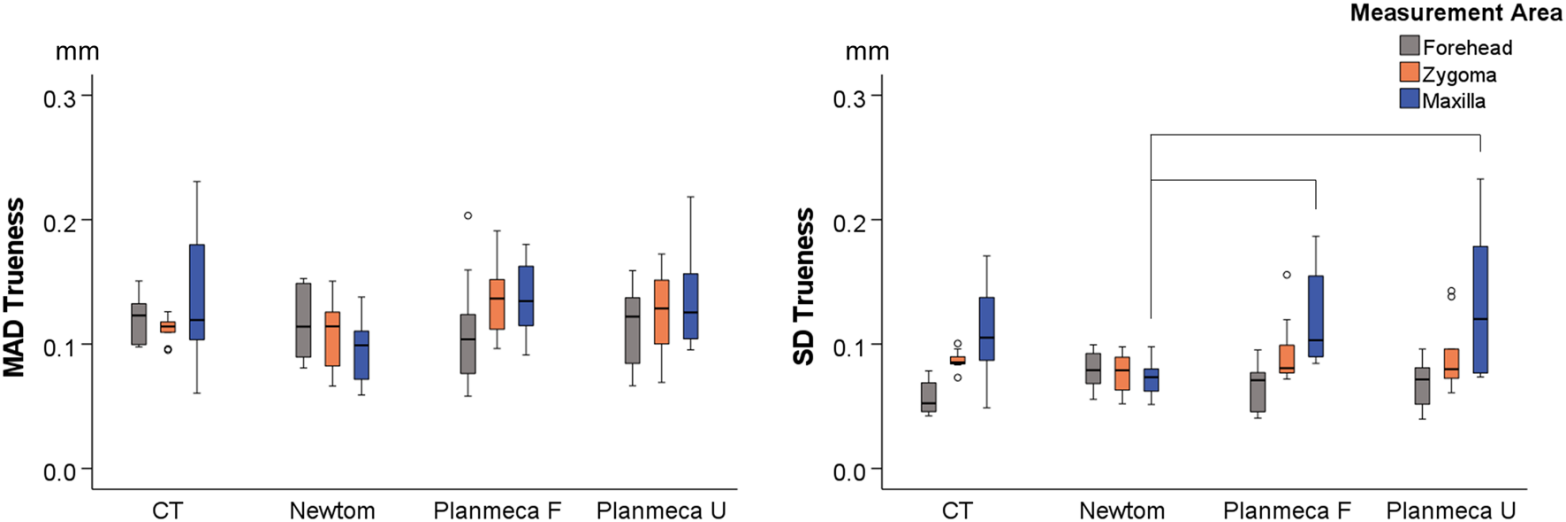
Box plots showing the trueness of the segmented facial surface models indicated by the distances of the radiographically derived models from the direct optical scans, following their best-fit approximation. The Mean Absolute Distances (MAD) and the standard deviations of the absolute distances (SD) between the superimposed surface models are shown. The lines connect variables that show significant differences (*p* < 0.05) detected through Kruskal–Wallis, followed by Mann–Whitney U test (Bonferroni adjusted). Outliers are shown as black circles (further from the median more than 1.5 times the IQR).

The overall trueness (MAD) was 0.117 mm (IQR: 0.045), with an SDAD of 0.080 mm (IQR: 0.028), with no difference (MAD) between acquisition settings, considering all measurement areas as one variable (Kruskal–Wallis Test: *p* = 0.118; CT, median: 0.116, IQR: 0.030; Newtom, median: 0.110, IQR: 0.051; Planmeca F, median: 0.125, IQR: 0.057; Planmeca U, median: 0.124, IQR: 0.053). Similarly, there was no difference on the SDADs between acquisition settings (Kruskal–Wallis Test, *p* = 0.407; CT, median: 0.084, IQR: 0.045; Newtom, median: 0.078, IQR: 0.027; Planmeca F, median: 0.083, IQR: 0.026; Planmeca U, median: 0.079, IQR: 0.046) (Fig. 7).

When each measurement area was analysed as a single variable, there was no difference in trueness (MAD of the best-fit approximated models) between acquisition settings. Similarly, for the SDAD very few, limited differences between acquisition settings were detected only at the maxilla (Newtom vs. Planmeca F: *p* = 0.013, Newtom vs. Planmeca U: *p* = 0.033) (Fig. 7).

**Figure 8:**
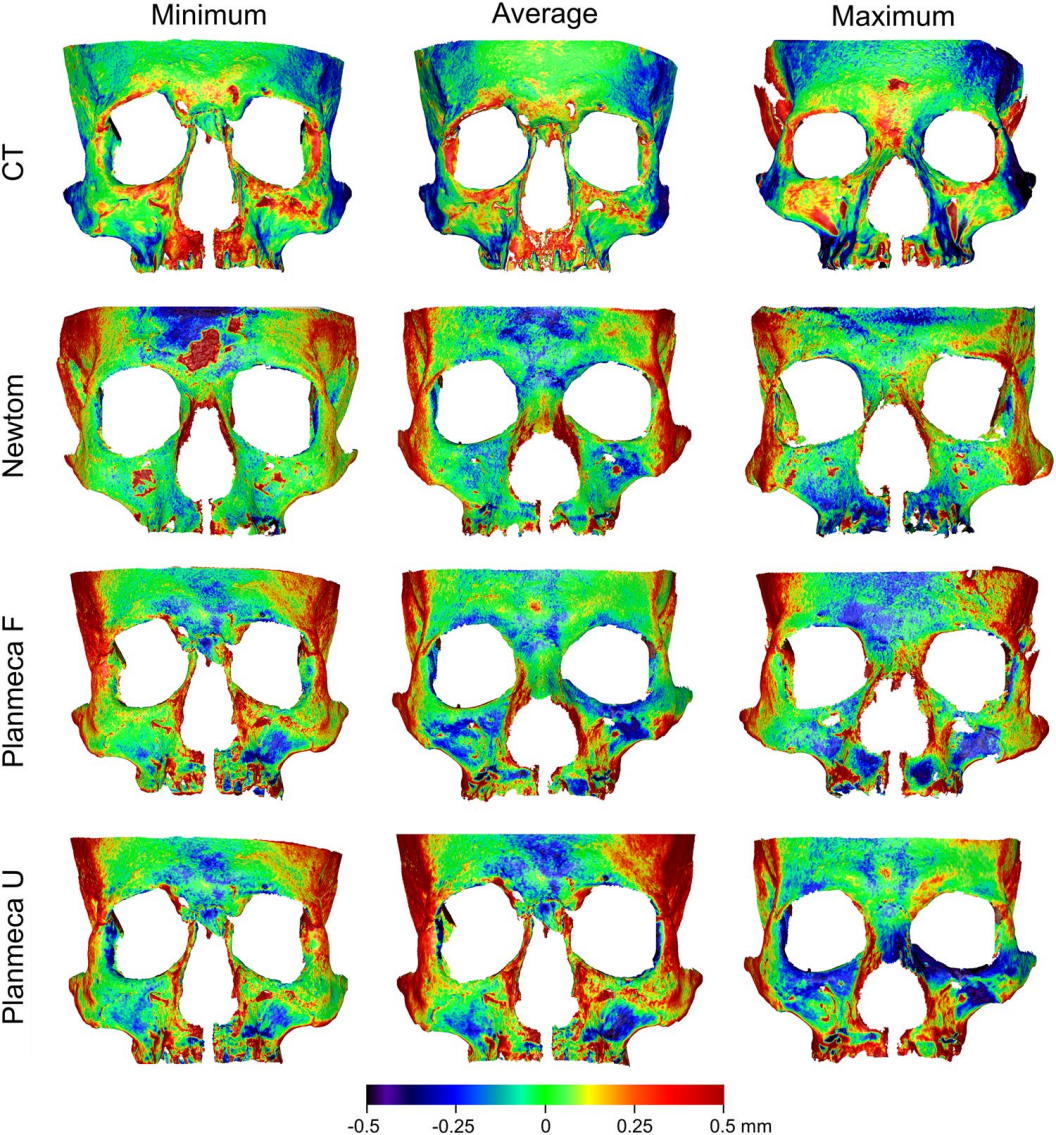
Colour coded distance maps between best-fit approximated segmented surface models and directly obtained models through an optical surface scanner, representative of the minimum, average, and maximum deviations in trueness, detected in the sample for each acquisition setting.

The colour coded distance maps between the best-fit approximated segmented and directly scanned facial surface models revealed consistent outcomes within and between acquisition settings, with deviations from the true model only locally reaching a maximum of 0.5 mm (Fig. 8). Large deviations were consistently present at the sides of the skull, located distant to the used superimposition reference area.

## Discussion

The present study tested the accuracy of radiographically derived 3D facial surface models against a gold standard reference model, applying advanced 3D image analysis methods. Previous studies attempted to test similar outcomes, albeit with certain shortcomings, such as the usage of artificially prepared models^27^, the lack of soft-tissue simulation^28–30^, or the use of inter-landmark distances as test outcomes^5,29^. Other studies highlighted these shortcomings and their impact on applicability issues^10,16,19,20,31,32^. We aimed to simulate real life conditions, by incorporating study design techniques that allowed for the simulation of soft-tissues^24^ and the acquisition of true reference models^22^, and we considered the entire facial surface for outcome assessment.

Although soft tissues have varying densities, and their representation in radiographic images may not be fully accurate with single material simulation, various materials have been shown to provide adequate soft-tissue simulation in similar experimental settings, including water, wax sheets, or gel-like materials^17,18,24^. We embedded the dry skulls in water during radiographic imaging to enable multiple scans without compromising the integrity of the specimens^20,22^. We hydrated the dry skulls similarly, prior to the true reference model acquisition by the high-accuracy optical scanner, since a previous study revealed that hydration alters the anatomical form of dry bones^20^.

This study tested the accuracy of both CT and CBCT radiographic scanners, using regular radiation dose settings and a low radiation setting, offered by one CBCT unit. The image acquisition protocols were defined according to the standard practice for the assessment of craniofacial morphology, by specialist radiologists that were regularly using the radiographic machines for clinical purposes. All tested methods performed similarly, exhibiting an average trueness of 0.12 mm, without any significant difference between machines or settings. Colour coded distance maps revealed local deviations extending up to 0.5 mm, but the overall models were robust in all cases, despite the different image acquisition configurations. This finding has significant implications when 3D surface models are sought for diagnosis, growth or treatment outcome evaluation, or applications such as virtual surgical planning and manufacturing of prostheses in maxillofacial surgery. In every circumstance, whether these accuracy levels are acceptable or not, depends on the specific research or clinical context in which they are intended to be applied. The present findings showed that imaging methods applying lower radiation than the conventional CT can provide adequate digital models for the assessment of facial morphology, without compromises in accuracy. This holds true even for the low dose acquisition with the Planmeca CBCT scanner, which was performed using half of the radiation dose of the regular scan by the same unit. In the same line, a previous qualitative study evaluating the visibility of selected anatomical structures of the jaws in large FOV CBCT images with lower exposure settings showed encouraging results^33^.

The overall trueness of 0.12 mm was very high when considering the magnitude of the optical scanner error^22^ and the segmentation error, at 0.03 mm and 0.07 mm, respectively. Larger deviations up to 0.5 mm were consistently present at the sides of the skull, which were distant to the selected superimposition reference area^3,10,22,34^, as well as to local sites over the entire tested surface, with no specific spatial pattern. The latter findings might be partially attributed to direct optical surface scanner inaccuracies that might sometimes exceed 0.2 mm locally^20,22^.

The segmentation reproducibility was lower for the CT derived surface models, when assessed at their original position in space. This difference can be attributed to how thresholding functions in each imaging modality. CT images use Hounsfield units, which is a standardized quantitative scale consistent throughout a dataset, meaning that two voxels with the same value correspond to the same tissue type and density. Therefore, slight changes in the threshold value may have a more distinct effect on the resulting 3D surface. On the other hand, grayscale values in CBCT do not have the same consistency, as they vary according to the surrounding structures of each target area and to its position in space. In CBCTs, small differences in the threshold values appeared to have a lesser effect. The voxel size differences might be another contributing factor, although it did not affect the trueness findings, even within the same machine (CT, Supplementary Fig. 3). In any case, the differences between repeatedly segmented models were consistently lower than 0.1 mm and the differences between repeatedly selected threshold values were consistently lower than 2% of the total range of values. Thus, any of the aforementioned factors had a minor effect on the reproducibility outcomes.

Although trueness was comparable among radiographic scanners and settings, its variance was higher for the CT and CBCT data from the Planmeca machine, especially in the maxillary areas. A possible explanation could be the porous structure of the maxilla in conjunction with the usage of a single threshold value for each dataset. The complex bone-air interface in this area may pose a challenge to depict accurately and reliably, when other factors such as the voxel size, FOV, and radiation dose are considered.

For the assessment of reproducibility and trueness, the left and right measurement areas were unified to consolidate homologous findings and facilitate the statistical analysis. This decision was based on previous studies that did not find differences between contralateral sides^3,10^. Moreover, we selected four circular surfaces for each measurement area to assess trueness, but only two for reproducibility. The reason is the higher resolution of the optical scanner as opposed to the radiographically derived 3D surfaces. Therefore, the gold standard reference models had a higher triangle count for a given area. To compensate for this difference, we performed four selections in each measurement area to assess trueness that had similar extent to the two analogous areas used for reproducibility testing.

### Strengths and limitations

Based on the outcomes of a previous study^20^, the hydration of the skulls has a significant effect on their geometric stability. This is important when investigating accuracy at a submillimetre level. Every specimen was embedded in water for 15 min prior to any image acquisition to closely simulate real life conditions, namely the presence of soft-tissues on radiographic images, and acquire directly comparable true reference models. Another strength is the inclusion of multiple widely used radiographic scanners to increase the study’s generalisability. The acquisition parameters were defined by radiology specialists to reflect imaging for actual clinical purposes, namely adequate diagnostic quality for the representation of actual anatomical morphology. Finally, the rigorous testing of the used hardware and software applications that provided the true models^20,22^, as well as of the 3D imaging, the superimposition, and the assessment methods, by our team, ensure the validity of the presented outcomes^2,3,10,23,34–36^.

A limitation of our study might be the usage of a single threshold value for each dataset. An alternative method is the manual segmentation for each anatomical region, but this is a very time consuming procedure that is still prone to error^37^. More recent, sophisticated approaches involving deep learning and artificial intelligence applications manage to reduce time considerably, but the segmentation accuracy levels are often not properly assessed or not substantially enhanced^38,39^. Due to its straightforward application, the single threshold segmentation remains the standard process for many imaging software, despite its weaknesses, especially in CBCT images. Here, both the intra- and inter-operator differences in the visual segmentation threshold values were small and did not differ between machines. Similar outcomes were evident in a recent study on anterior cranial base surface models, where the visually defined single threshold value showed very small differences from the manually defined^2^. The repeatedly extracted facial surface models consistently showed differences lower than 0.1 mm in the entire facial surface. With this straightforward approach, the present study showed high accuracy outcomes, despite the weaknesses stemming from the CBCT image characteristics, where the grayscale intensity values do not directly correspond to the tissue type and density throughout the entire radiographic volume. The latter, combined with the large extent of the assessed surface models, was expected to affect negatively the outcomes, since the optimal threshold might differ between anatomical areas of the same tissue type and density that are located at different positions in the radiographic volumes. Regardless, the accuracy outcomes were robust throughout the tested facial surfaces. It should be mentioned that when imaging actual patients, motion artefacts may lead to greater inaccuracies^40^. Moreover, the software’s algorithm measures the distances between selected vertices on one surface and the closest vertices on the other surface, which might not necessarily be anatomically correspondent. This minimizes the differences between the two approximated surface meshes, and thus, the actual anatomical differences may be slightly larger. Finally, the age that the tested skulls represented was not known. It is expected that most of the skulls, if not all, would belong to adult subjects. As a result, we should anticipate a specific age range, but this cannot be further defined. However, the absence of this information did not impact the comparisons within the study, as the same skulls were utilized across all methods. The outcomes were robust with no outliers, but still no solid conclusions about children can be drawn from the present study.

This study focused on facial surface models, which comprise an important anatomical area for several fields^5,10,12^. Other areas, with thin cortical bone, such as the mandibular condyles or the anterior cranial base, which might be hard to segment in large field of view scans^2,19^, were not assessed. That would require the application of newly designed assessment protocols, including bone segmentation procedures, and could be the topic of future studies. Future research should also focus on the assessment of craniofacial morphology in three dimensions, while minimizing patient exposure to radiation. The fact that the tested low radiation protocol showed comparable accuracy to the standard protocols is encouraging. A decrease of the field of view and the proper adjustment of other scanning parameters can lead to further reduction of radiation exposure^23,33,41^.

## Conclusions

The repeatedly extracted facial surface models through a visually defined single threshold showed consistently differences lower than 0.1 mm over the entire facial surface, for all acquisition configurations. All CT and CBCT radiographic images exhibited an average trueness of 0.12 mm, with local deviations reaching 0.5 mm in certain cases. There were no significant differences between radiographic scanners or settings and the inaccuracies were evenly distributed over the entire facial surface. These findings are of high significance establishing the accuracy of lower radiation CBCT imaging at a similar level to the conventional CT images. Furthermore, a lower radiation CBCT protocol was shown to perform adequately and similarly to the standard CBCT imaging protocols in the depiction of the actual skeletal facial morphology, shifting the cost/benefit ratio of a given acquisition to a more favourable level for our patients.

### Supplementary Information

Supplementary Figures.

## Data Availability

All data are available in the main text or the extended data. The datasets generated and/or analyzed during the current study will be available on request from the corresponding author. Due to the sensitive nature of the used specimens, the raw data would remain confidential and would not be shared.

## References

[1] Ye H (2022). Comparison of the accuracy (trueness and precision) of virtual dentofacial patients digitized by three different methods based on 3D facial and dental images. J. Prosthet. Dent..

[2] Friedli L, Kloukos D, Kanavakis G, Halazonetis D, Gkantidis N (2020). The effect of threshold level on bone segmentation of cranial base structures from CT and CBCT images. Sci. Rep..

[3] Kanavakis G, Ghamri M, Gkantidis N (2022). Novel anterior cranial base area for voxel-based superimposition of craniofacial CBCTs. J. Clin. Med..

[4] Halazonetis DJ (2005). From 2-dimensional cephalograms to 3-dimensional computed tomography scans. Am. J. Orthod. Dentofac. Orthop..

[5] Dings JP (2019). Reliability and accuracy of cone beam computed tomography versus conventional multidetector computed tomography for image-guided craniofacial implant planning: An in vitro study. Int. J. Oral Maxillofac. Implants.

[6] Oh SH (2018). Linear accuracy of cone-beam computed tomography and a 3-dimensional facial scanning system: An anthropomorphic phantom study. Imaging Sci. Dent..

[7] Pauwels R, Araki K, Siewerdsen JH, Thongvigitmanee SS (2015). Technical aspects of dental CBCT: State of the art. Dentomaxillofac. Radiol..

[8] Schulze R (2011). Artefacts in CBCT: A review. Dentomaxillofac. Radiol..

[9] Ghoneima A, Cho H, Farouk K, Kula K (2017). Accuracy and reliability of landmark-based, surface-based and voxel-based 3D cone-beam computed tomography superimposition methods. Orthod. Craniofac. Res..

[10] Haner ST, Kanavakis G, Matthey F, Gkantidis N (2020). Voxel-based superimposition of serial craniofacial CBCTs: Reliability, reproducibility and segmentation effect on hard-tissue outcomes. Orthod. Craniofac. Res..

[11] Jamróz W, Szafraniec J, Kurek M, Jachowicz R (2018). 3D printing in pharmaceutical and medical applications - recent achievements and challenges. Pharm. Res..

[12] de Lima Moreno JJ, Liedke GS, Soler R, da Silveira HED, da Silveira HLD (2018). Imaging factors impacting on accuracy and radiation dose in 3D printing. J. Maxillofac. Oral Surg..

[13] Luan F-J, Zhang J, Mak K-C, Liu Z-H, Wang H-Q (2021). Low radiation X-rays: Benefiting people globally by reducing cancer risks. Int. J. Med. Sci..

[14] Liang X (2010). A comparative evaluation of cone beam computed tomography (CBCT) and multi-slice CT (MSCT). Part II: On 3D model accuracy. Eur. J. Radiol..

[15] van Leeuwen BJ (2022). Effect of voxel size in cone-beam computed tomography on surface area measurements of dehiscences and fenestrations in the lower anterior buccal region. Clin. Oral Investig..

[16] Dusseldorp JK, Stamatakis HC, Ren Y (2017). Soft tissue coverage on the segmentation accuracy of the 3D surface-rendered model from cone-beam CT. Clin. Oral Investig..

[17] Caldas MP, Ramos-Perez FMM, Almeida SM, Haiter-Neto F (2010). Comparative evaluation among different materials to replace soft tissue in oral radiology studies. J. Appl. Oral Sci..

[18] Lopes PA, Santaella GM, Lima CAS, Vasconcelos KF, Groppo FC (2019). Evaluation of soft tissues simulant materials in cone beam computed tomography. Dentomaxillofac. Radiol..

[19] García-Sanz V (2017). Accuracy and reliability of cone-beam computed tomography for linear and volumetric mandibular condyle measurements. A human cadaver study. Sci. Rep..

[20] Dritsas K (2022). Effect of hydration on the anatomical form of human dry skulls. Sci. Rep..

[21] Lindsten R (2002). The effect of maceration on the dental arches and the transverse cranial dimensions: A study on the pig. Eur. J. Orthod..

[22] Probst J (2022). Precision of a hand-held 3D surface scanner in dry and wet skeletal surfaces: An ex vivo study. Diagnostics (Basel).

[23] Gkantidis N (2015). Evaluation of 3-dimensional superimposition techniques on various skeletal structures of the head using surface models. PLoS One.

[24] Wang X (2011). Material separation in x-ray CT with energy resolved photon-counting detectors. Med. Phys..

[25] Lorensen WE, Cline HE (1987). Marching cubes: A high resolution 3D surface construction algorithm. ACM Siggr. Comput. Graphics.

[26] Besl PJ, Mckay ND (1992). A method for registration of 3-D shapes. IEEE Trans. Pattern Anal. Mach. Intell..

[27] Matta R-E (2016). The impact of different cone beam computed tomography and multi-slice computed tomography scan parameters on virtual three-dimensional model accuracy using a highly precise ex vivo evaluation method. J. Cranio-Maxillofac. Surg..

[28] Kang S-H, Kim M-K, Kim H-J, Zhengguo P, Lee S-H (2014). Accuracy assessment of image-based surface meshing for volumetric computed tomography images in the craniofacial region. J. Craniofac. Surg..

[29] Lorkiewicz-Muszyńska D (2015). Accuracy of the anthropometric measurements of skeletonized skulls with corresponding measurements of their 3D reconstructions obtained by CT scanning. Anthropol. Anz..

[30] Probst FA (2021). Geometric accuracy of magnetic resonance imaging-derived virtual 3-dimensional bone surface models of the mandible in comparison to computed tomography and cone beam computed tomography: A porcine cadaver study. Clin. Implant. Dent. Relat. Res..

[31] Mai DD, Stucki S, Gkantidis N (2020). Assessment of methods used for 3-dimensional superimposition of craniofacial skeletal structures: A systematic review. PeerJ..

[32] Utermohle CJ, Zegura SL, Heathcote GM (1983). Multiple observers, humidity, and choice of precision statistics: Factors influencing craniometric data quality. Am. J. Phys. Anthropol..

[33] Baumann E, Bornstein MM, Dalstra M, Verna C, Dagassan-Berndt DC (2022). Image quality assessment of three cone beam computed tomography scanners-an analysis of the visibility of anatomical landmarks. Eur. J. Orthod..

[34] Ghamri M, Kanavakis G, Gkantidis N (2021). Reliability of different anterior cranial base reference areas for voxel-based superimposition. J. Clin. Med..

[35] Häner ST, Kanavakis G, Matthey F, Gkantidis N (2021). Valid 3D surface superimposition references to assess facial changes during growth. Sci. Rep..

[36] Winkler J, Sculean A, Gkantidis N (2022). Intraoral scanners for in vivo 3D imaging of the gingiva and the alveolar process. J. Clin. Med..

[37] Wang L (2014). Automated bone segmentation from dental CBCT images using patch-based sparse representation and convex optimization. Med. Phys..

[38] Issa J, Olszewski R, Dyszkiewicz-Konwińska M (2022). The effectiveness of semi-automated and fully automatic segmentation for inferior alveolar canal localization on CBCT scans: A systematic review. Int. J. Environ. Res. Public Health.

[39] Yeshua T (2023). Deep learning for detection and 3D segmentation of maxillofacial bone lesions in cone beam CT. Eur. Radiol..

[40] Birklein L (2023). Motion correction for separate mandibular and cranial movements in cone beam CT reconstructions. Med. Phys..

[41] Vogiatzi T, Menz R, Verna C, Bornstein MM, Dagassan-Berndt D (2022). Effect of field of view (FOV) positioning and shielding on radiation dose in paediatric CBCT. Dentomaxillofac. Radiol..

