# Supplementary material for: Accuracy of facial skeletal surfaces segmented from CT and CBCT radiographs
Source: Sci Rep. 2023 Nov 28;13:21002. doi: 10.1038/s41598-023-48320-0 (PMC10684569; doi:10.1038/s41598-023-48320-0)
Supplement: Supplementary file 1 — Supplementary Figures. [file 41598_2023_48320_MOESM1_ESM.pdf]

Supplementary information

Accuracy of facial skeletal surfaces segmented from CT and CBCT radiographs.

Mohammed Ghamri, Konstantinos Dritsas, Jannis Probst, Maurus Jäggi, Symeon Psomiadis, Ralf Schulze, Carlalberta Verna, Christos Katsaros, Demetrios Halazonetis, Nikolaos Gkantidis

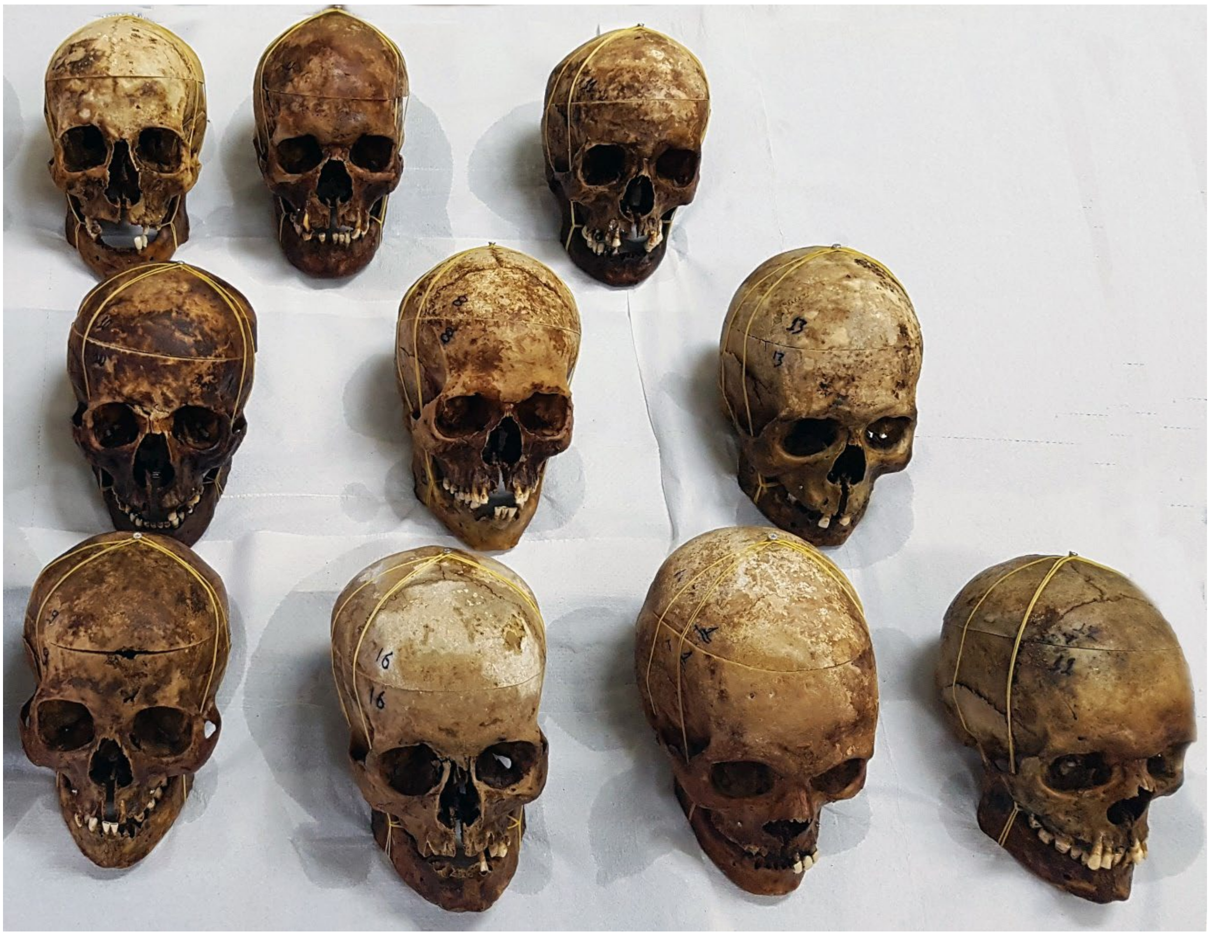

Supplementary Figure 1. Ten human dry skulls that were used in the study. The skulls were embedded in water as shown in the image. The count of present teeth is not indicative of age, given that teeth that were fully functional during an individual's lifetime are frequently lost post-mortem due to tissue degradation.

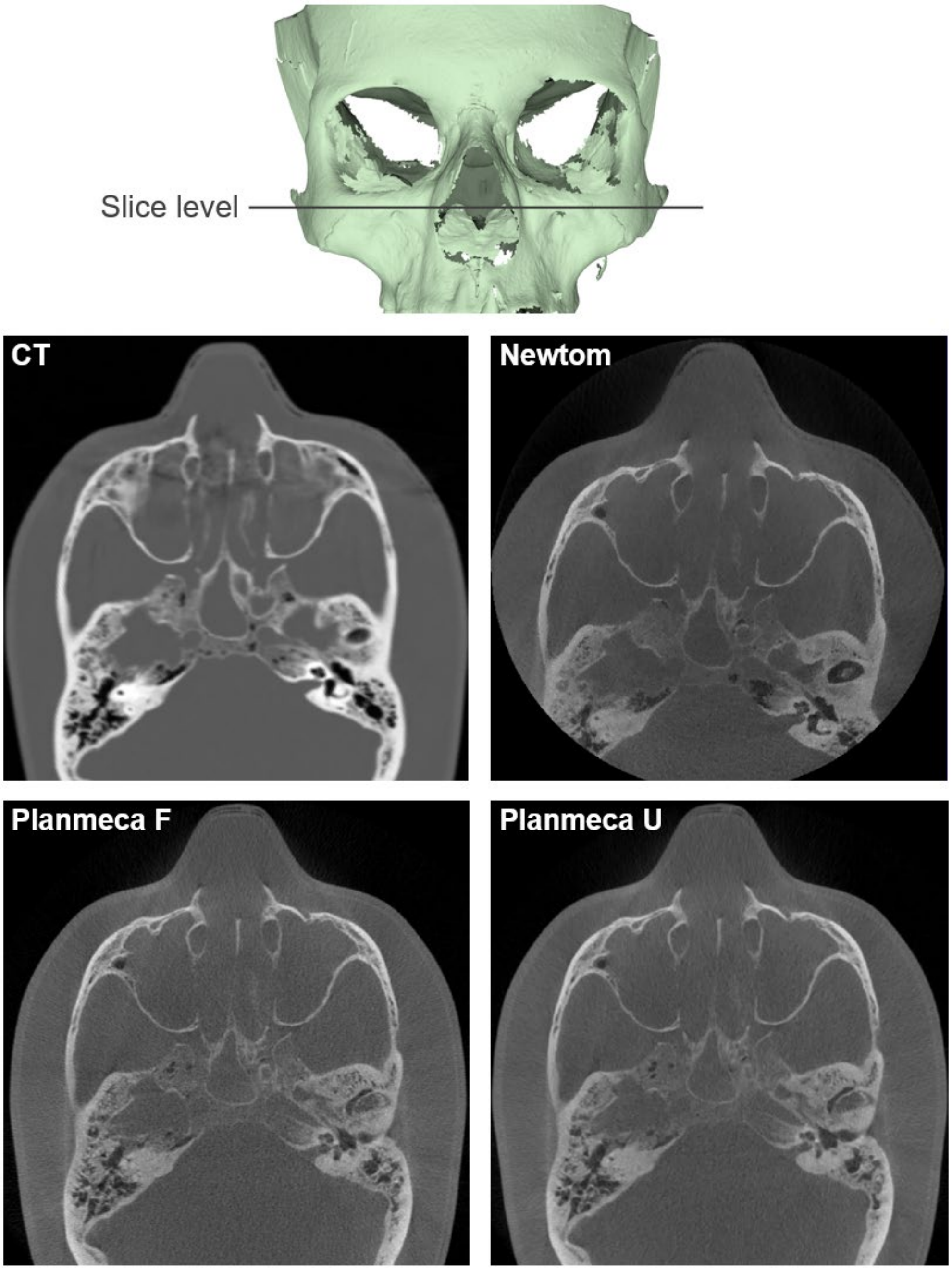

Supplementary Figure 2. Sample transversal images of radiographic volumes obtained from the same skull, through each acquisition setting, at the level of the zygomatic arch.

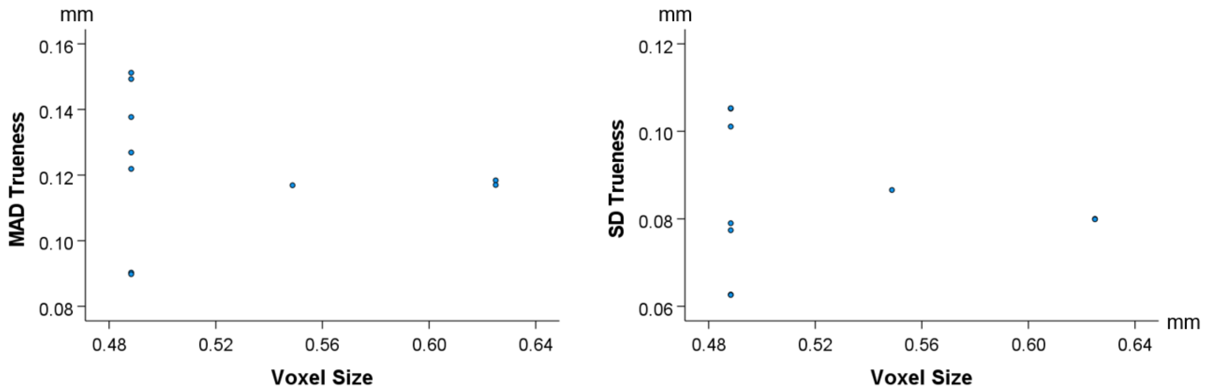

Supplementary Figure 3. Scatter dot plots of the voxel size in CT radiographic volumes against the trueness values. The Mean Absolute Distances (MAD) and the standard deviations of the absolute distances (SD) between the best-fit approximated radiographically derived and directly scanned surface models are the measured variables shown. There was no correlation between the plotted variables (MAD, Spearman’s rho = -0.315, p = 0.376; SD, Spearman’s rho = 0.090, p = 0.805).
